# Therapeutic Vaccines for Genitourinary Malignancies

**DOI:** 10.3390/vaccines6030055

**Published:** 2018-08-12

**Authors:** Giselle M. A. Dutcher, Mehmet Asim Bilen

**Affiliations:** 1 Department of Medicine, Emory University School of Medicine, Atlanta, GA 30322, USA; gdutche@emory.edu; 2 Winship Cancer Institute, Emory University, Atlanta, GA 30322, USA

**Keywords:** prostate cancer, renal cell cancer, bladder cancer, cancer immunotherapy, therapeutic cancer vaccine, combination therapies

## Abstract

The field of genitourinary malignancies has been a showcase for therapeutic cancer vaccine success since the application of intravesicular Bacillus Calmette-Guerin (BCG) for bladder cancer in the 1970s and enjoyed a renaissance in 2010 with the US Food and Drug Administration (FDA) approval of sipuleucel-T for prostate cancer. Several vaccine strategies have emerged, such as autologous or allogeneic whole-tumor vaccines, DNA vaccines, use of viral vectors, and peptides as immunostimulatory adjuvants. Despite impressive early trials, vaccine monotherapy has achieved limited success in the clinical world; however, combinations of vaccine and immune checkpoint inhibition or vaccine and cytokine stimulation are expected to move the field forward. This article reviews pivotal trials of cancer vaccines in prostate, renal, and bladder cancer and ongoing trials combining vaccines with other immune therapy agents.

## 1. Introduction

Therapeutic cancer vaccines are an important part of the larger approach of immunotherapy for cancer treatment, which aims to bolster the immune system to recognize and eliminate tumor cells. The success of ipilimumab in metastatic melanoma [1] ushered in the era of immune checkpoint antibodies to programmed cell death protein (PD1) and its ligand (PDL1) that have changed the landscape of treatment for advanced solid tumors. Ongoing research seeks to amplify the immune effect with agonist antibodies to costimulatory receptors [2]. More recently, immune therapy has achieved clinical benefit in liquid malignancies with the approval of chimeric antigen receptor T-cell (CAR-T) therapy for refractory acute lymphoblastic leukemia [3] and chronic lymphocytic leukemia [4].

The field of genitourinary malignancies has been a showcase for therapeutic cancer vaccine success since the application of intravesicular Bacillus Calmette-Guerin (BCG) for bladder cancer in the 1970s [5] and enjoyed a renaissance in 2010 with the US Food and Drug Administration (FDA) approval of sipuleucel-T for prostate cancer [6]. Several vaccine strategies have emerged, such as autologous or allogeneic whole-tumor vaccines, DNA vaccines, use of viral vectors, and peptides as immunostimulatory adjuvants [7,8]. In addition to their elegant mechanism of action, vaccine strategies are clinically valuable for having a low adverse event profile. Particularly in advanced disease, patients who are not candidates for curative therapies may benefit from a vaccine therapy or combination that keeps tumors in an indolent stage [8]. These approaches all aim to increase antigen production to overcome antigen evasion in the tumor, or in other words, to generate a heated immune attack on immunologically cold tumors.

## 2. Prostate Cancer 

### 2.1. Sipuleucel-T

Sipuleucel-T is the first and only FDA-approved vaccine therapy for metastatic, castration-resistant prostate cancer (mCRPC). This vaccine is an example of personalized therapy—the vaccine is made from a patient’s own antigen-presenting cells (APC) activated with PAP2024, a fusion protein of prostatic antigen phosphatase and granulocyte-macrophage colony-stimulating factor (GM-CSF) [9,10]. IMPACT—the landmark trial leading to the approval of sipuleucel-T in 2010—showed that sipuleucel-T monotherapy increased overall survival (OS) by four months compared to placebo [6]. This finding of increased OS, while modest, was notable because the vaccine therapy was very well tolerated and because docetaxel chemotherapy was the only therapy at the time with overall survival benefit for metastatic prostate cancer [11]. Initial analyses indicated that in real-world use, patients with lower baseline prostate-specific antigen (PSA) who received sipuleucel-T had better OS [12]. In another retrospective analysis, patients with older age and high tumor burden (>20 bone metastasis and high alkaline phosphatase) were more likely to experience rapid progression [13]. These findings suggest that vaccine therapy has greater benefit when given early in the course of metastatic prostate cancer. At present, sipuleucel-T is less commonly used in clinical practice due to lack of biomarkers to correlate with response (no imaging or PSA response), challenging administration due to plasmapheresis, and the approval of novel antiandrogen therapies (such as enzalutamide and abiraterone) [14,15,16,17]. However, due to the excellent side effect profile, sipuleucel-T may be a useful addition as a combination treatment.

Current studies are investigating the combination of sipuleucel-T with androgen deprivation therapy. STAMP—a phase II study (NCT 01487863) of sipuleucel-T with abiraterone in combination versus sequentially—showed no difference in APC activation between the two groups, suggesting that concurrent therapy does not blunt the immune response [18]. A subsequent report of long-term outcomes in the STAMP cohort [19] showed median OS of 34 months and median time to progressive disease of 17.3 months. The cohort was not large enough to detect a significant difference in outcomes between groups. A small phase II trial (STAND) has provided some evidence for sequencing of therapy; a greater antitumor response was seen with sipuleucel-T prior to androgen deprivation therapy in 68 patients [20]. However, prospective trials to assess the clinical outcomes are yet to be determined. Lastly, a cytokine-based approach to immune stimulation is the most recent combination. Interleukin agonist CYT107 stimulates IL7 and has been shown to promote T-cell recovery after stem cell transplantation [21] and in metastatic breast cancer patients with leukopenia [22]. Given that sipuleucel-T depends on T-cell response, CYT107 is an attractive booster; currently, a phase II study (see Table 1) of CYT107+sipuleucel-T in asymptomatic mCRPC is underway.

### 2.2. Other Vaccines—GVAX, PROSTVAC, and pVTG-HP

PROSTVAC-VF—a peptide-based vaccine targeting PSA [23]—showed great promise in early trials. In a phase II study, PROSTVAC-VF showed an increase in OS of eight months compared with the placebo group (empty vector) [14]. Unfortunately, the phase III trial PROSPECT was terminated early when preliminary analyses showed no effect on OS [24]. The authors concluded that the vaccine alone was not sufficient to overcome the low immunogenicity in prostate cancer.

Whole-cell vaccines theoretically provide multiple antigen targets for immune activity. This approach was tried with GVAX, a whole-cell vaccine consisting of two prostate cancer cell lines made to express GM-CSF [25]. Combined phase I/II trials showed good tolerability, increased antibody response with increasing doses, and PSA response in 20% of the patients, suggesting clinical efficacy [25,26]. On the basis of these promising results, two phase III trials were opened. VITAL-1 compared GVAX versus docetaxel; although fully enrolled, the trial was terminated when interim analysis indicated futility in meeting the primary endpoint [27]. Similarly, VITAL-2, which compared GVAX alone versus GVAX plus docetaxel, was terminated after interim analysis showed increased deaths in the treatment group. The GVAX trials again showed that vaccine monotherapy could not match the survival benefit of docetaxel, and in the absence of a specific biomarker, immune response and clinical benefit could not be determined.

DNA vaccines are another attractive approach for anticancer immunotherapy [28]. Of these, the vaccine pVTG-HP, which contains plasmid DNA encoding prostatic acid phosphatase (PAP) to elicit PAP-specific T-cells, has progressed furthest in development. Safety and immunological efficacy was shown in 22 patients with biochemical recurrent prostate cancer who received six doses [29,30]. PAP-specific CD4 and CD8 T-cell responses were observed in 41% of patients, and no significant adverse effects were noted [30]. A subsequent pilot study of vaccine schedule confirmed long-term PAP-specific immune response with repeated immunization over 24 weeks; furthermore, 38% of patients remained free of metastatic disease [29]. Based on this data, a phase II trial is underway to determine the effect of pVTG-HP monotherapy on metastasis-free survival in patients with early castrate-resistant disease (Table 2). Combination therapy with other agents may increase the efficacy of vaccines in advanced castrate-resistant disease.

### 2.3. Vaccines in Combination (Docetaxel, Androgen Deprivation Therapy, and Immune Checkpoint Inhibitors)

A phase II study with 28 patients investigated the T-cell response from a recombinant peptide-based PSA vaccine (similar to PROSTVAC) and docetaxel concurrently versus vaccine alone [31], and it showed no difference in T-cell response at three months. This small study was not powered for analysis of progression-free survival (PFS) or OS, but the median time to progression (biochemical or radiographic) was greater in the subset of patients who received docetaxel after progression on the vaccine (6.2 months) compared to those who received docetaxel and vaccine concurrently (3.2 months) [31]. The time to progression in the vaccine alone group was 1.8 months, suggesting that combination therapy with docetaxel (whether concurrent or subsequent) improves the clinical response. A follow-up trial was designed to assess OS in patients randomized to docetaxel alone versus PROSTVAC followed by docetaxel. Unfortunately, this trial closed due to insufficient accrual—only 10 patients of a planned 144 were enrolled. The authors’ analysis highlights the difficulty of enrolling patients for a study when several treatment options are available [32]. Furthermore, the authors highlight the importance of validated biochemical endpoints (evaluation of T-cell response, PSA velocity, or PSA doubling time) that should be used consistently across phase II studies to facilitate comparison.

Initial studies of nivolumab [33] and ipilimumab [34] as single agent therapy for advanced prostate cancer did not show a survival benefit. However, rapid PSA reduction and partial response have been reported in a small cohort treated with pembrolizumab [35], which encourages further study of immune checkpoint inhibitors. Since prostate cancer is considered an immunologically “cold” tumor, vaccine therapy becomes an attractive partner to stimulate antigen response.

Several studies combining vaccination with checkpoint inhibitors are currently underway; these are summarized in Table 1. The phase I trial for PROSTVAC+ipilimumab showed no increased toxicity and suggested a survival benefit for combination therapy in comparison to historical controls [36]. Similar trials are underway for PROSTVAC+nivolumab and GVAX+ipilimumab (Table 1). Optimal dosing and sequence are not yet determined. A small phase I trial of sipuleucel-T with escalating doses of ipilimumab suggested better response at higher doses [37]. An ongoing phase II trial is assessing whether ipilimumab should be given immediately or delayed compared to starting sipuleucel therapy (Table 1). The DNA vaccine pVTG-HP in combination with pembrolizumab is being studied in an ongoing phase II trial. Data from a pilot trial of this combination showed antitumor response in patients with concurrent, but not sequential, therapy [38].

## 3. Renal Cell Cancer

Renal cell carcinoma (RCC) is known to be an “immune active tumor.” The first therapies showing survival benefit for metastatic RCC—interleukin-2 (IL-2) and interferon-alpha (IFN)—were approved in the 1990s, before the current immune checkpoint era [8]. However, IL-2 and IFN have high toxicity, which has limited their use to patients with good performance status [39]. In the last 10 years, several additional therapies have arisen for first-line or subsequent treatment. Targeted therapies include tyrosine kinase inhibitors (TKI) cabozantinib, sorafenib, sunitinib, and pazopanib; and mammalian target of rapamycin (mTOR) inhibitors everolimus and temsirolimus [40,41,42,43,44]. Recently, immune checkpoint inhibitors have shown significant OS improvement in RCC. Initially, nivolumab was approved as single agent front-line therapy for poor-risk RCC patients [45]. In 2018, the combination of ipilumumab+nivolumab was approved in front-line RCC treatment for intermediate and poor-risk patients [46]. Concurrently, vaccine approaches are also under development, with IMA091 and AGS003 being the two most relevant.

### 3.1. IMA091

IMA091—an off-the-shelf vaccine composed of 10 different tumor associated peptides—had very promising results in early clinical trials, showing robust immune response [47]. In the phase II trial of 68 patients randomized 1:1 to vaccine alone versus cyclophosphamide+vaccine, immune response was similar between the two groups. Patients who received cyclophosphamide priming had a trend to improved survival. Importantly, immune response was associated with clinical outcome—patients with immune response had longer survival compared to non-responders. Of note, the primary outcome of disease control rate (complete response + partial response + stable disease) was twice as good in patients with previous cytokine treatment than in patients with prior TKI therapy [48].

Following those impressive early trials, a large phase III trial of 339 patients in multiple centers (IMPRINT) was designed to study the combination of vaccine IMA091+GMCSF+cyclophosphamide priming+sunitinib versus sunitinib alone [49]. Despite high expectations, IMPRINT did not show improved OS in the vaccine group. The patients were followed for nearly three years, and at the study conclusion, there was no statistical difference in OS of 33 months in the vaccine group vs. not reached in the control group. Specifically, 50% of patients in the combination group died compared to 40% in the sunitinib alone group. Additionally, the T-cell responses in the phase III study were three times lower than in the phase I and II studies, and the T-cell response was not associated with clinical outcome [49]. The low immune response from the vaccine is problematic; the investigators feel this must be enhanced prior to additional studies or combination therapies.

### 3.2. AGS003

A slightly different approach is seen with AGS003, a personalized vaccine manufactured from patient’s dendritic cells and patient’s tumor RNA in addition to synthetic CD40L. In a phase II trial, AGS003 was given after the first cycle of sunitinib and continued for five boosters or until disease progression. The trial closed enrollment early but still reported results of 21 patients [50]. There were no complete responses, but 13 patients experienced clinical benefit (nine partial response and four stable disease). Median OS from registration was 30 months, with 12 patients surviving beyond four years. In addition, good T-cell responses were seen, and the increase in absolute number of T-cells correlated with survival [50].

A further study of AGS was the ADAPT trial, a phase III trial that compared AGS+sunitinib to sunitinib alone. ADAPT completed enrollment of 450 patients and although interim analysis in February 2017 recommended trial discontinuation due to expected futility, the trial investigators decided to continue the study [51]. The ADAPT investigators felt that additional time was necessary to see a difference between the groups as median duration of follow-up was only 20 months and over half the patients in each group were still living. A post-hoc subgroup analysis showed median OS of 30 months in the combination arm versus 22 months with sunitinib alone [52]. Additional immunology data were presented in November 2017 showing statistically significant increase in antigen-specific T-cells compared to baseline and correlation with improved survival [53]. However, in April 2018, the ADAPT trial was terminated when additional analysis did not show a benefit of the combination vaccine+sunitinib treatment [54].

Since the design of the IMPRINT and ADAPT studies, checkpoint inhibitors have risen to front-line therapy for poor-risk, advanced RCC. Although checkpoint inhibitors have expanded the treatment arsenal, optimal therapy and sequencing are yet to be clarified. For those patients who do not respond to checkpoint inhibitors, vaccine combination might be a valid strategy to either prime or prolong the immune response. Further studies are warranted before vaccines for renal cell cancer are dismissed.

## 4. Bladder Cancer

Intravesicular BCG has been used for the treatment of bladder cancer since the 1970s and is the accepted first-line treatment for nonmuscle invasive bladder cancer (NMIBC) after resection [5]. The mechanism is not well understood, but the vaccine is thought to activate cytokines and T-cell recruitment leading to an immune memory [55]. Treatment options for muscle invasive bladder cancer had been limited to platinum-based chemotherapy until the recent accelerated FDA approval of immunotherapy agents atezolizumab [56] and pembrolizumab [57] for patients ineligible for or with progression on platinum-based chemotherapy. Subsequently, durvalamab [58], nivolumab [59], and avelumab [60] were also approved for this population. While several trials are ongoing for front-line use of immunotherapy agents in the muscle invasive setting, in May 2018, the FDA released a safety alert calling attention to decreased survival in patients with low PD-L1 expression who received atezolizumab or pembrolizumab as first-line monotherapy [61].

### Combinations with Intravesicular BCG and Novel Vaccine Approaches

Combination of immunotherapy with vaccines remains an important strategy; several ongoing trials are summarized in Table 3. For NMIBC typically treated with intravesicular BCG alone, the combination of immunotherapy may lead to better response for patients at high risk of disease recurrence. Early phase trials with pembrolizumab and atezolizumab are currently active. Cytokine stimulation is also being studied in bladder cancer with ALT-803, an agent that stimulates IL-15 and enhances cytotoxic T-cells. In the phase Ib study of ALT-803 with intravesicular BCG, patients with NMIBC remained disease-free at 12 months, and no grade 3/4 adverse events were reported [62]. The combination of intravesicular BCG and ALT-803 shows great promise for NMIBC, such that this combination was granted fast-track status by the FDA [63]. While BCG remains a mainstay of bladder cancer treatment, other vaccine approaches are also being considered.

Cancer neoantigens are peptides unique to cancer cells resulting from tumor mutations. With the advent of tumor sequencing, these peptides can be identified and used as targets for a personalized anticancer vaccine. This approach is currently being investigated in a phase I trial of melanoma, lung, and bladder cancer with the combination of NEO-PV-01 (vaccine) and nivolumab [64]. In this phase I study, participants will receive nivolumab first, then the vaccine treatment. Bladder cancer patients with prior BCG therapy are eligible; the expected study completion is 2020. 

## 5. Conclusions

Although there has been no FDA approval of a cancer vaccine since sipuleucel-T for prostate cancer in 2010, the field is far from stagnant. The trials for prostate and kidney cancer vaccines highlighted in this review—PROSTVAC, GVAX, AGS, and IMA901—showed promise in early phases but did not have the expected clinical outcomes when given as monotherapy. In prostate cancer, there is renewed interest in trials of vaccines with immune checkpoint inhibitors. Vaccines for renal cell carcinoma were not successful as monotherapy when compared to targeted therapy; however, given the success of checkpoint inhibitors, vaccines deserve another chance in a combination approach. The success of immune therapy for bladder cancer began with BCG monotherapy for nonmuscle invasive disease and has expanded to the treatment of advanced disease thanks to immune checkpoint inhibitors and cytokines.

## Figures and Tables

**Table 1:** Ongoing trials of immunotherapy combinations in prostate cancer.

Approach	NCT ID	Therapy	Population	Phase	Summary
Dual vaccine	01706458	Sipuleucel T+pVTG-HP (DNA booster vaccine)	Asymptomatic mCRPC	II	Active
Vaccine+ADT	01867333	PROSTVAC+enzalutamide vs. enzalutamide alone	mCRPC	II	Active, not recruiting
Vaccine+checkpoint inhibitor	02506114	PROSTVAC monotherapy, ipilumumab monotherapy, or combination therapy (both PROSTVAC and ipilumumab)	Localized prostate cancer, treatment-naive, prior to radical prostatectomy	II	Ongoing
	02499835	DNA Vaccine MVI-816+pembrolizumab	mCRPC, progression on ADT	II	Ongoing
	01832870	Sipuleucel T+escalating dose of ipilimumab	mCRPC	I	Increased PAP and PA2024 immunoglobulins
	01804465	Sipuleucel T+immediate versus delayed ipilumumab	mCRPC	II	Ongoing
	02933255	PROSTVAC+nivolumab	mCRPC	I/II	Ongoing
	00113984	PROSTVAC+ipilumumab	mCRPC	I	Safe, no dose-limiting toxicity
	01510288	GVAX+ipilumumab	mCRPC	I	Safe, no dose-limiting toxicity for ipilumumab 3 mg/kg
Vaccine+Interleukin agonist	01881867	Sipuleucel T+CYT107	Asymptomatic mCRPC	II	CYT107 stimulates IL7

**Table 2:** Important trials of vaccine monotherapy in prostate cancer.

Study Name NCT ID	Therapy	Result
IMPACT [3]	Sipuleucel-T Personalized vaccine (autologous APC+PAP)	FDA approval
PROSPECT [14]	PROSTVAC (vaccine targeting PSA peptide)	Terminated due to futility
VITAL-1 NCT00089856 VITAL-2 NCT00133224	GVAX (whole tumor vaccine) versus docetaxel GVAX+docetaxel versus GVAX monotherapy	Terminated due to futility Terminated due to safety
pTVG-HP NCT01341652	pTVG-HP (DNA vaccine encoding PAP)	Active/In progress

**Table 3:** Ongoing vaccine trials in bladder cancer.

Approach	NCT ID	Intervention	Population	Phase	Summary
BCG plus checkpoint inhibitor	02792192	Intravesicular BCG+atezolizumab versus atezolizumab alone	High-risk nonmuscle invasive bladder cancer	Ib/II	Pharmacokinetics study, escalating BCG dose, fixed atezolizumab dose
	02808143	Intravesicular BCG+Pembrolizumab	Nonmuscle invasive bladder cancer	I	Pharmacokinetics study, escalating pembrolizumab dose
Vaccine+checkpoint inhibitor	02897765	NEO-PV-01+adjuvant (Poly-ICLC)+nivolumab	Metastatic bladder cancer	Ib	Personalized neoantigen vaccine
Vaccine only	03132922	MAGE-A4^c1o32^ T cell therapy	Bladder cancer expressing MAGE-A4 protein	I	Autologous genetically engineered Tcells targeting MAGE-A4
Vaccine+BCG	02010203	Intravesicular BCG+HS-410 vaccine	Nonmuscle invasive bladder cancer	I/II	Irradiated cancer cells engineered to produce heat shock protein gp96 which stimulate CD8 Tcell signaling
BCG+ Interleukin agonist	02138734	Intravesicular BCG+ALT-803	Nonmuscle invasive bladder cancer	I	Pharmacokinetics, determine maximum tolerated dose of ALT-803
	03022825	Intravesicular BCG+ALT-803	Nonmuscle invasive bladder cancer with failure of BCG treatment	II	ALT-803 received fast track status by FDA
